# Aberrant TTF-1 expression in metastatic colorectal adenocarcinoma mimicking primary lung cancer: a case report and review of diagnostic pitfalls

**DOI:** 10.1186/s13000-025-01732-0

**Published:** 2025-11-27

**Authors:** Nhu Tung Tran, Nguyen Van Thai, Nguyen Thi Khuyen, Linh Thi Le, Lai The Anh Khoa

**Affiliations:** 1 Department of Pathology, Tam Anh General Hospital, Ho Chi Minh City, Vietnam; 2 Vietnam National Cancer Hospital, Hanoi, Vietnam; 3 Department of Radiology, An Binh Hospital, Ho Chi Minh City, Vietnam

## Abstract

A 43-year-old man with sigmoid colon adenocarcinoma (low-grade, moderately) developed multiple pulmonary metastases, presenting an unusual immunohistochemical profile. Histologically, resected lung nodules showed metastatic adenocarcinoma consistent with colorectal origin, yet the tumor cells paradoxically expressed thyroid transcription factor-1 (TTF-1) – a marker typically specific to primary lung adenocarcinoma. Immunophenotyping demonstrated TTF-1 nuclear positivity in the metastatic tumor alongside a classic colorectal profile: cytokeratin 7 (CK7) negativity, cytokeratin 20 (CK20) positivity, strong caudal-type homeobox transcription factor 2 (CDX2) and special AT-rich sequence-binding protein 2 (SATB2) nuclear expression, and absence of Napsin A. The patient underwent surgical resection of the primary sigmoid colon tumor and received 16 cycles of capecitabine plus bevacizumab chemotherapy. Molecular testing revealed a KRAS c.35G > T (p.G12V) mutation in the tumor. This case highlights a potential diagnostic pitfall in metastatic colorectal cancer: aberrant TTF-1 expression can mimic a primary lung tumor. We discuss how the comprehensive immunohistochemical panel and genetic findings confirmed the colorectal origin of the lung lesions, emphasizing that combined marker profiles (TTF-1 +/CK7 –/CK20 +/CDX2 +/SATB2 +/Napsin A –) are more consistent with metastatic colorectal adenocarcinoma rather than an enteric-type adenocarcinoma of the lung, primary. The report reviews relevant literature and underscores the importance of correlating clinical history with pathology to avoid misdiagnosis.

## Introduction

Colorectal carcinoma (CRC) frequently metastasizes to the lung, where it may closely resemble primary pulmonary adenocarcinoma. This distinction is clinically crucial, as misclassification can lead to inappropriate therapy. Immunohistochemistry (IHC) is central to resolving this dilemma: CRC typically shows CK7 negativity with CK20, CDX2, and SATB2 positivity, whereas primary lung adenocarcinomas are usually TTF-1 and CK7 positive, with Napsin A expression [1, 2].

TTF-1 is widely regarded as a lung (and thyroid) lineage marker, expressed in ~ 73–90% of pulmonary adenocarcinomas and generally absent in gastrointestinal tumors. However, it is not entirely specific: rare cases of adenocarcinomas from the colon, breast, ovary, endometrium, and even the central nervous system have demonstrated unexpected TTF-1 positivity. Rare reports have documented aberrant TTF-1 expression in CRC, particularly when the SPT24 antibody clone is used, creating a diagnostic pitfall [3, 4].

Here, we present a case of a 43-year-old male with sigmoid colon adenocarcinoma metastatic to the lungs, in which the metastases exhibited an otherwise typical colorectal immunoprofile but with the unexpected feature of TTF-1 nuclear positivity. We detail the clinical history, radiologic findings, pathology, IHC workup, and molecular results, and discuss the diagnostic implications of this rare phenomenon in the context of current literature.

## Methods

**Fig. 1:**
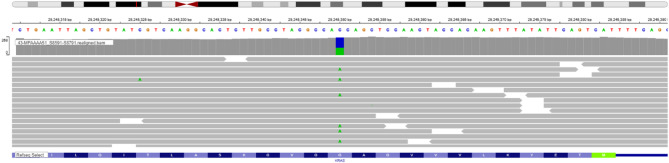
KRAS mutation c.35G > T (p.G12V)

A 43-year-old man with no significant medical or family history presented with intermittent abdominal pain and altered bowel habits. Clinical evaluation revealed no recognized colorectal cancer risk factors, including hereditary syndromes (Lynch, familial adenomatous polyposis, MUTYH-associated polyposis), inflammatory bowel disease, prior colorectal polyps, metabolic disorders, or smoking and heavy alcohol use. Colonoscopy revealed a sigmoid mass, and biopsy with subsequent surgical resection confirmed invasive adenocarcinoma. The tumor invaded through the muscularis propria into pericolonic fat with lymphovascular invasion; two of 25 regional lymph nodes were positive (pT3N1, AJCC 8th Edition). The malignancy was mismatch-repair proficient by immunohistochemistry and carried a KRAS c.35G > T (p.G12V) mutation (Fig. 1).

**Fig. 2:**
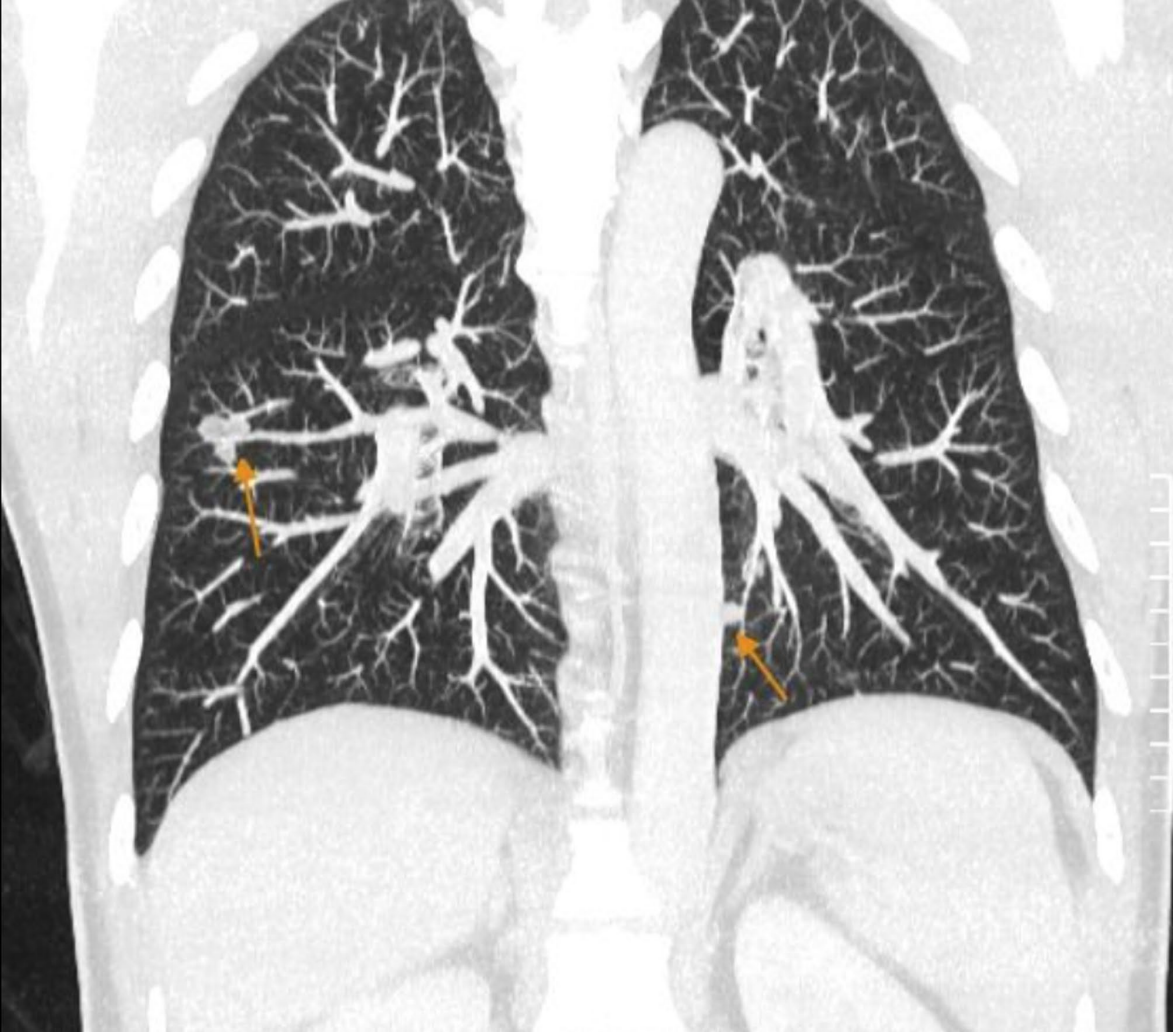
CT chest scan showing bilateral pulmonary nodules

Staging contrast-enhanced CT scans of the chest, abdomen, and pelvis revealed no liver lesions but showed multiple bilateral pulmonary nodules measuring 7–10 mm in diameter, suspicious for metastases. The lung nodules were distributed in both upper and lower lobes without a dominant mass, consistent with hematogenous metastatic spread (Fig. 2**)**. No other distant metastases were identified. After recovery from colon surgery, the patient underwent a wedge resection of a representative right lung nodule for diagnostic confirmation. Histopathology of the wedge resection is detailed below.

Axial contrast-enhanced chest CT shows seven solid nodules, measuring 7–10 mm, with attenuation of 30–40 HU and moderate contrast uptake. The nodules are scattered in the upper, middle, and lower lobes bilaterally, consistent with hematogenous metastases.

Following resection of the primary tumor, the patient received systemic chemotherapy combined with targeted therapy. He was treated with 16 cycles of capecitabine (oral fluoropyrimidine) plus bevacizumab (an anti-VEGF monoclonal antibody) as a first-line regimen for metastatic colorectal cancer. This regimen was chosen in lieu of oxaliplatin-based therapy due to patient preference and to avoid neurotoxicity. Treatment was well tolerated, with the main side effects being hand-foot syndrome and hypertension, which were managed medically. Interim imaging during therapy showed stable pulmonary disease with no new lesions. After completing 16 cycles, a CT scan demonstrated radiologic stability of the lung nodules, consistent with a partial response/controlled disease. The patient was then placed on surveillance with follow-up imaging. At the time of this report (approximately 18 months from initial diagnosis), he is alive and under close observation; the pulmonary metastases remain stable in size with no evidence of new metastatic spread.

## Results

### Histopathology and immunohistochemistry

**Fig. 3:**
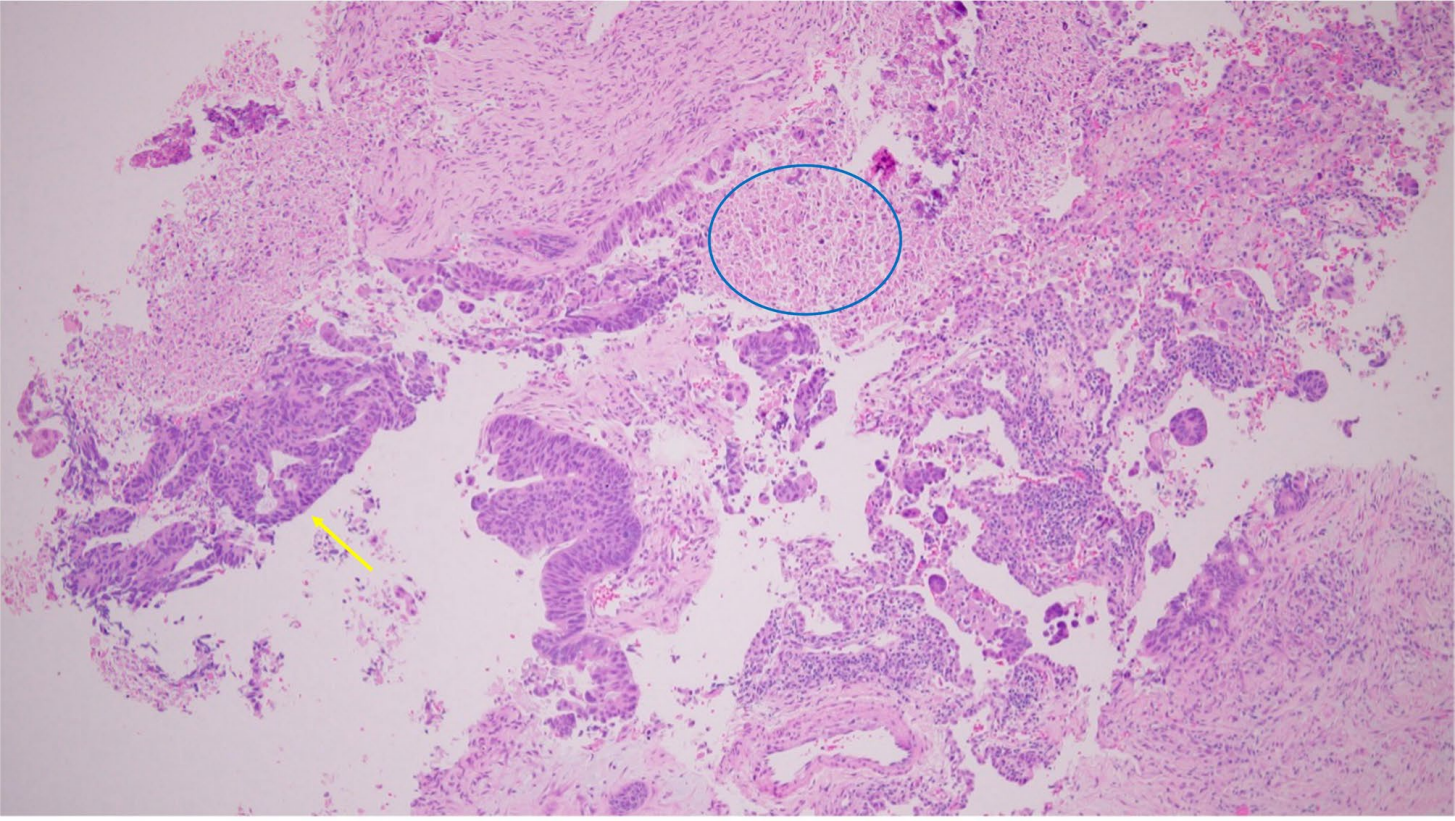
H&E section of the lung nodule showing metastatic adenocarcinoma with irregular malignant glands (yellow arrows) and intraluminal necrotic debris (“dirty necrosis,” blue oval); original magnification ×100

Hematoxylin and eosin (H&E)-stained section of the resected lung nodule shows metastatic adenocarcinoma infiltrating the lung parenchyma. The tumor forms irregular malignant glands, consistent with metastatic colorectal origin. The glands are lined by atypical columnar cells with elongated hyperchromatic nuclei, moderate pleomorphism, and mitotic activity. The neoplastic glands showed central luminal necrosis with granular debris (dirty necrosis), a feature commonly observed in colorectal adenocarcinomas **(**Fig. 3**).**

**Fig. 4:**
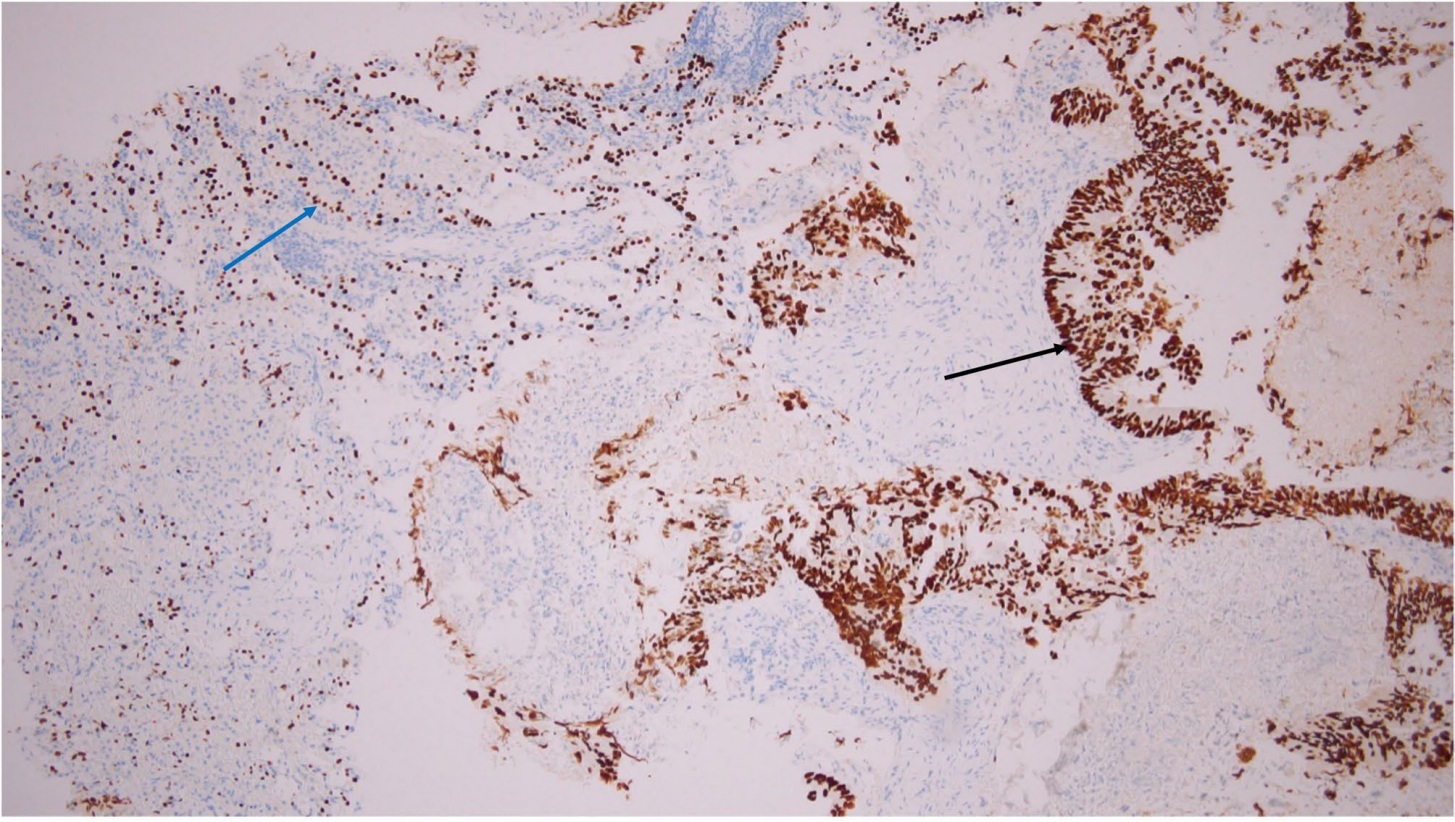
IHC for TTF-1 in lung metastasis showing strong nuclear positivity in tumor cells (black arrows). Residual type II pneumocytes serve as an internal positive control with nuclear TTF-1 expression (blue arrow); original magnification ×100

Remarkably, the tumor cell nuclei show distinct brown chromogen staining for TTF-1,confirming TTF-1 positivity in the metastatic colorectal adenocarcinoma.Internal positive controls (normal lung) are also present: the nuclei of residual type II pneumocytes in uninvolved alveoli show TTF-1 positivity (Fig. 4).

**Fig. 5:**
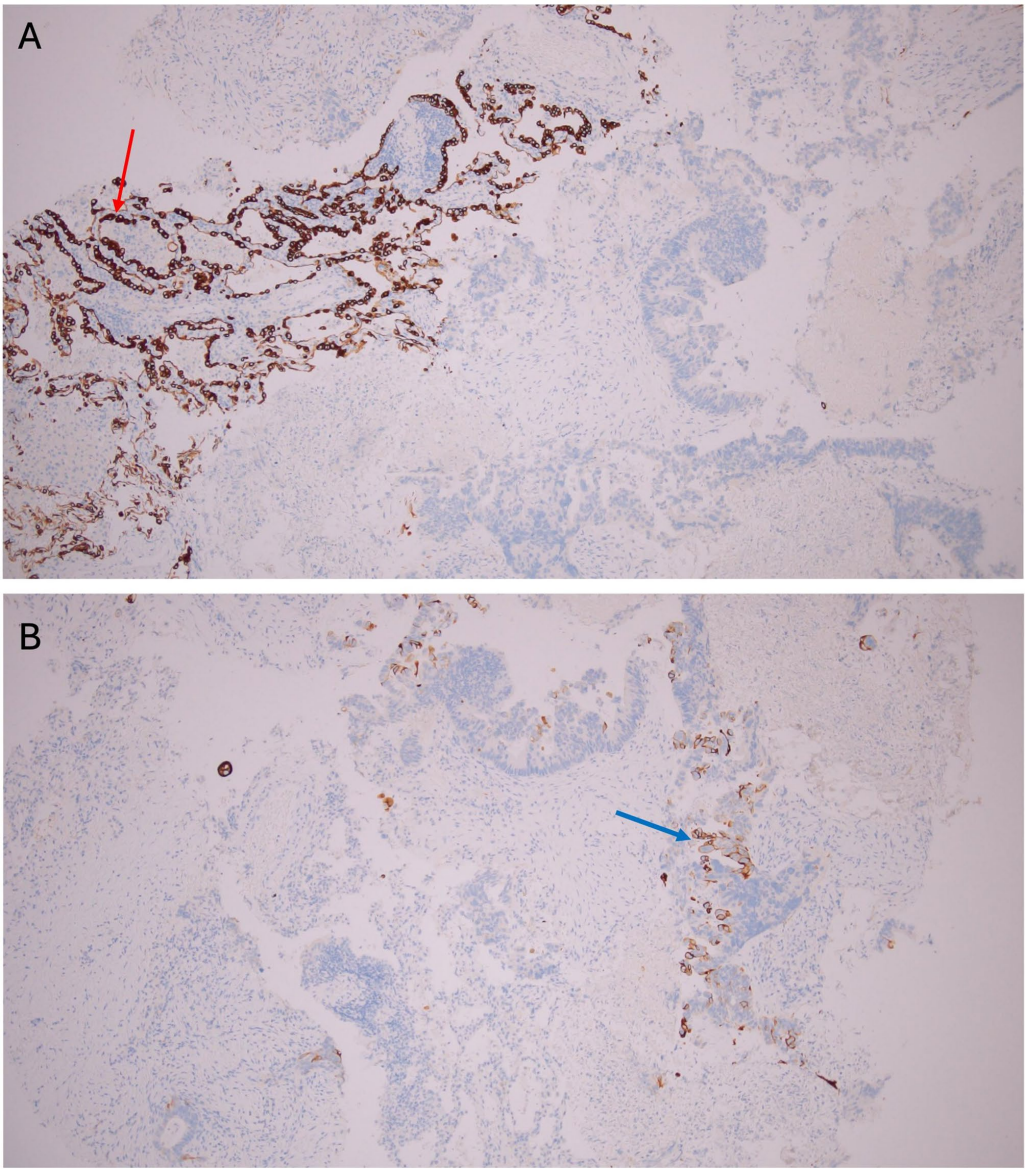
**A** IHC showing tumor cells negative for CK7, with residual pneumocytes as internal positive controls (red arrow); original magnification ×100. **B** Tumor cells showing positivity for CK20 (blue arrow); original magnification×100

**Fig. 6:**
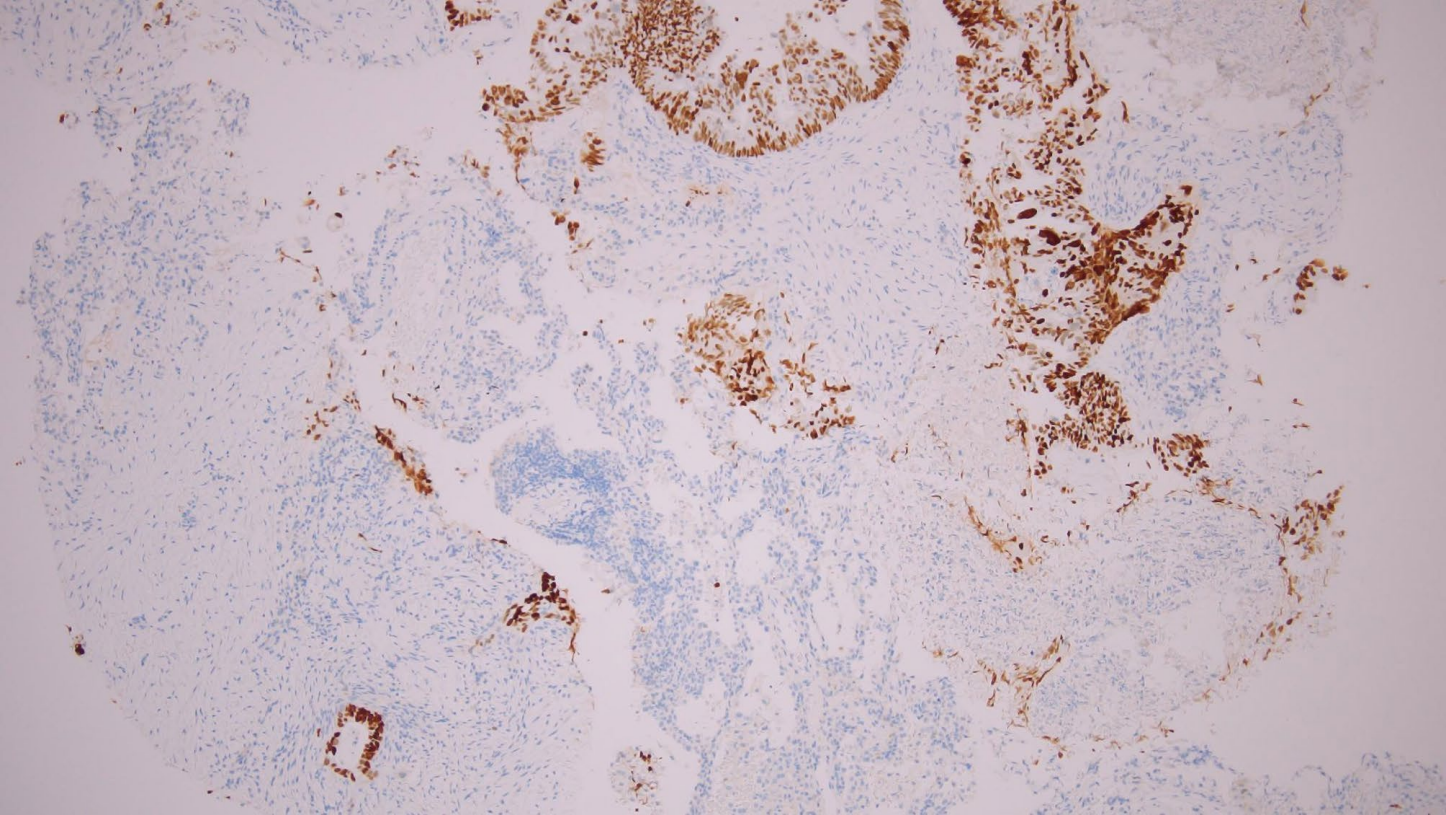
IHC showing nuclear positivity for CDX2; original magnification ×100

**Fig. 7:**
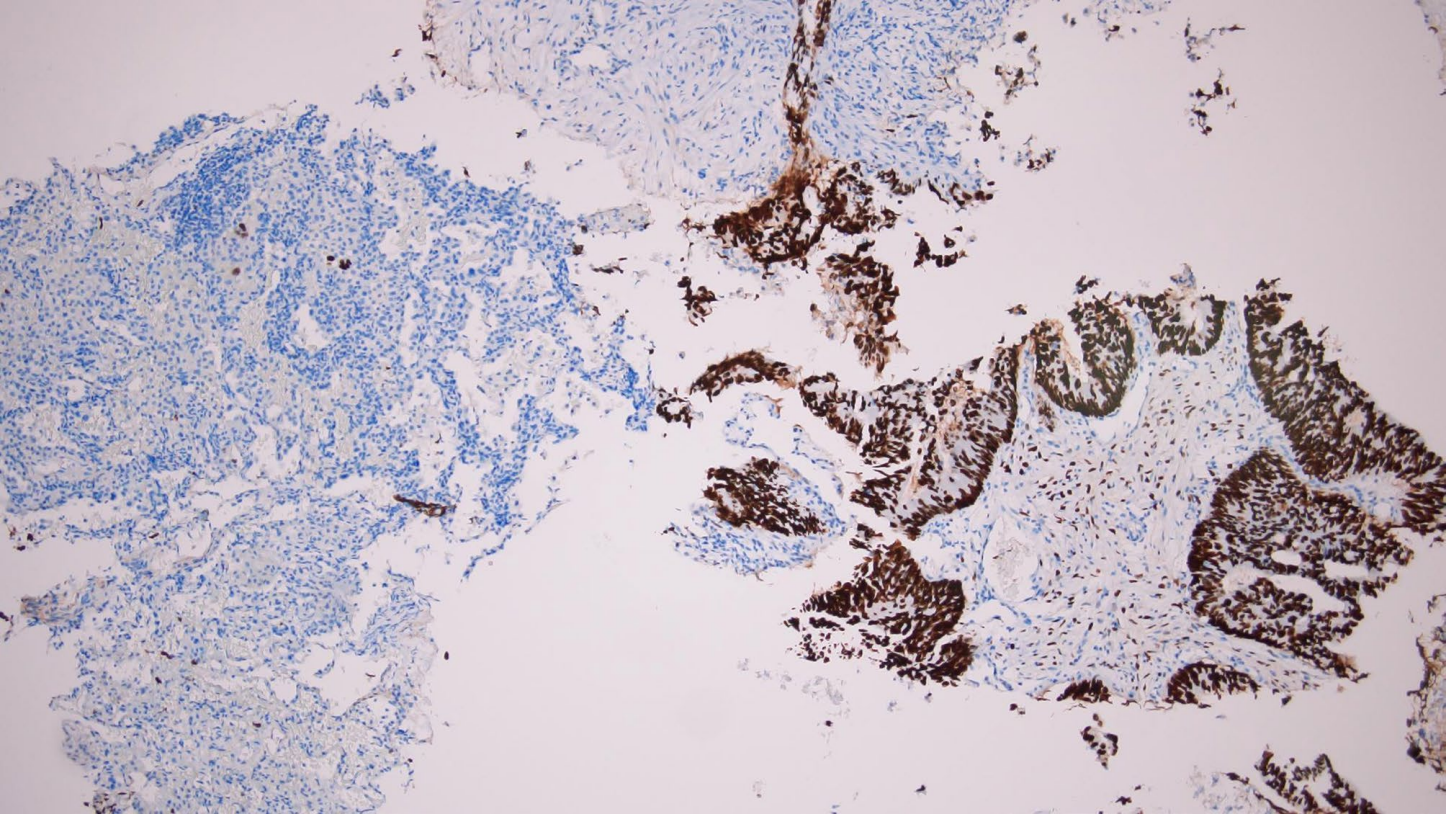
IHC showing nuclear positivity for SATB2; original magnification ×100

Immunohistochemistry supported a colorectal origin. CK7 was completely negative in tumor cells, with residual pneumocytes providing internal positive control (Fig. 5A). CK20 showed cytoplasmic staining in the neoplastic glands, absent in the surrounding lung tissue (Fig. 5B). CDX2 demonstrated diffuse nuclear positivity in nearly all tumor cells, confirming intestinal differentiation (Fig. 6). SATB2 was likewise strongly and diffusely positive in tumor nuclei, a highly specific feature of colorectal lineage and rarely seen in pulmonary adenocarcinoma (Fig. 7). Taken together, the profile (CK7–/CK20+/CDX2+/SATB2+) was diagnostic of metastatic colorectal adenocarcinoma, overriding the unexpected TTF-1 positivity.

Taken together, the histopathology and IHC profile (summarized in Table 1) confirmed that the multifocal lung tumors represented metastatic colorectal adenocarcinoma, despite the misleading TTF-1 positivity.

**Table 1:** Immunohistochemical profile of the lung metastasis compared to expected profiles of colorectal vs. lung adenocarcinoma [1, 2, 5–8]

Marker	Lung Metastasis (Patient’s tumor)	Colorectal Adenocarcinoma	Lung Adenocarcinoma
TTF-1	Positive	Negative (rarely positive in ~ 3–6% of cases)	Positive 73–90; strong diffuse in many)
CK7	Negative	Negative (>78%)	Positive (∼80–100% cases)
CK20	Positive	Positive (diffuse in >84% of most)	Rarely positive (< 5% cases)
CDX2	Positive	Positive (diffuse nuclear in >95% cases)	Rarely positive (< 10% cases; often focal)
SATB2	Positive	Positive (85–93%; high specificity for CRC)	Very rarely positive (∼1%)
Napsin A	Negative	Negative (100%)	Positive (∼80% cases)

## Discussion

**Fig. 8:**
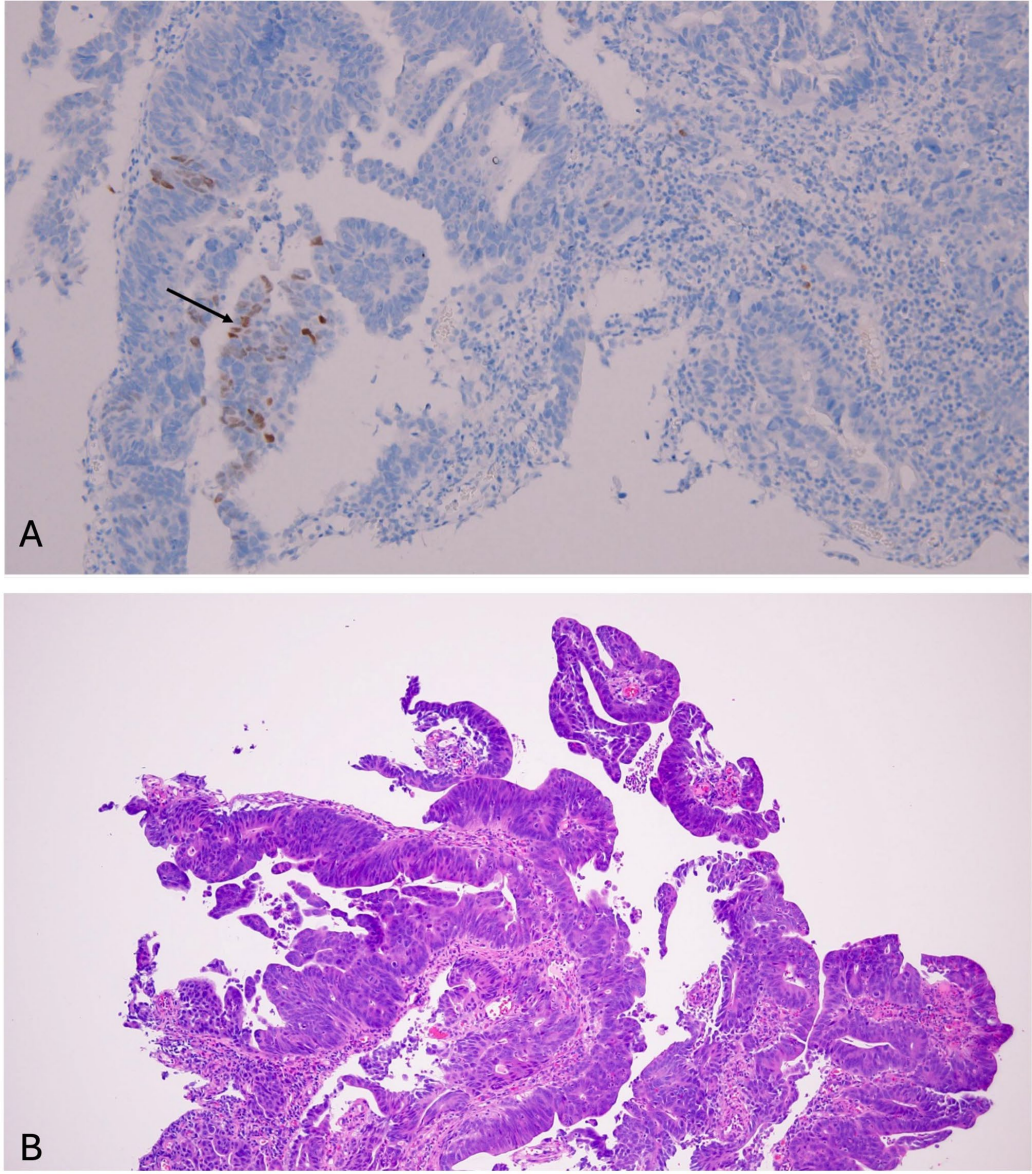
**A** IHC of the primary colon tumor showing focal nuclear TTF-1 staining in 1–5% of tumor cells (black arrow); original magnification ×200. **B** H&E section of the primary tumor; original magnification ×100

This case illustrates a rare but important diagnostic pitfall: metastatic colorectal adenocarcinoma to the lung with aberrant TTF-1 expression. TTF-1 is highly sensitive for primary lung adenocarcinoma (positive in about 70–90% of cases) but not entirely specific. Large series have shown that approximately 3–6% of colorectal carcinomas may also express TTF-1, particularly with the SPT24. Fewer than 20 cases of TTF-1–positive colorectal adenocarcinoma metastases to the lung have been documented, making our case noteworthy; importantly, all TTF-1-positive cases co-expressed CDX2 and nearly all were CK20-positive7. These findings imply that ectopic TTF-1 expression, while rare, is an intrinsic property of a subset of CRCs rather than an artifact of the metastatic lung environment8. In fact, retrospective immunostaining of the original colon tumor block (performed after the metastatic focus was found to be TTF-1 positive) showed patchy TTF-1 nuclear staining in the primary tumor as well (focally 1–5% of cells, Fig. 8), confirming that the primary colon cancer itself aberrantly expressed TTF-111.

Given this diagnostic pitfall, TTF-1 should never be interpreted in isolation when evaluating a lung tumor in patients with known colorectal cancer. Our case showed the classic colorectal immunoprofile (CK7–/CK20+/CDX2+/SATB2+) despite TTF-1 positivity, a constellation essentially diagnostic of metastatic CRC. SATB2 is particularly valuable, as it is positive in ~ 90% of CRCs but rare in lung. Napsin A negativity further supported a non-pulmonary origin. The major alternative differential diagnosis, enteric-type adenocarcinoma of the lung, usually retains CK7 expression and shows SATB2 only rarely, whereas our case was CK7–/SATB2+, excluding this possibility. The presence of a KRAS G12V mutation in the context of colorectal carcinoma is common – KRAS is mutated in approximately 40% of CRCs, with codon 12 substitutions (especially G12D and G12V) being the most prevalent [9]. In lung adenocarcinomas, KRAS mutations also occur (about 20–30%, often in smokers), but the most frequent subtype in lung cancer is G12C. The identification of the same KRAS mutation in both the colon primary and the lung metastasis confirmed the clonal relationship between the two. From a therapeutic standpoint, the KRAS mutation (especially at codon 12) also explained why anti-EGFR therapy was not employed; KRAS-mutant CRCs are resistant to EGFR inhibitors, so the patient was managed with chemotherapy and bevacizumab instead [9–11].

## Conclusion

We report a rare case of TTF-1–positive metastatic colorectal adenocarcinoma to the lung in a 43-year-old man, illustrating a diagnostic pitfall. Although the lung lesions expressed the lung-associated marker TTF-1, their complete immunohistochemical profile, along with the clinical history, clearly indicated a colorectal origin. This case highlights the need for a broad IHC panel and careful clinicopathologic correlation, as a small subset of colorectal cancers can aberrantly express TTF-1. Integration of histology, immunophenotype, and molecular findings confirmed metastatic colorectal carcinoma and guided appropriate colorectal cancer–directed therapy. The case also highlights the importance of SATB2 in differentiating colorectal metastases from primary lung tumors.

## Data Availability

No datasets were generated or analysed during the current study.
